# Quantification of Workload and Wellness Measures in a Women's Collegiate Volleyball Season

**DOI:** 10.3389/fspor.2021.702419

**Published:** 2021-08-06

**Authors:** Natalie Kupperman, Michael A. Curtis, Susan A. Saliba, Jay Hertel

**Affiliations:** Department of Kinesiology, University of Virginia, Charlottesville, VA, United States

**Keywords:** volleyball, accelerometer, workload, wellness, jumps

## Abstract

The purpose of this paper was to quantify internal and external loads completed by collegiate volleyball athletes during a competitive season. Eleven players were sampled (using accelerometers and subjective wellness surveys) during the practice (*n* = 55) and game (*n* = 30) sessions over the 2019 season. Longitudinal data were evaluated for trends across the preseason, non-conference play, and conference play periods. Data were also analyzed with respect to positional groups. Longitudinal analysis of accelerometer data showed higher workload demand during practices than games. Positional group differences were most when evaluating jump count and height. Setters accrued over twice as many jumps in a practice than during a game and had similar overall jump counts in practice to attacking positions. Average team wellness values varied with time in the season, especially during times of congested travel. This is the first study to look at both game and practice workload and wellness measures in collegiate women's volleyball. The results suggest athlete monitoring can be used to understand the demands of volleyball and used in the future to enhance practice and recovery day design to optimize athlete well-being.

## Introduction

Global positioning systems (GPS) and accelerometer technologies have become widespread in sports such as soccer, rugby, and football over the past decade as another way to quantify athlete workload. In recent years, these technologies have emerged in other sports as well, including the sport of volleyball. According to the American Volleyball Coaches Association, 96% of National Collegiate Athletic Association (NCAA) schools sponsor a women's volleyball team, translating into approximately 17,000 collegiate players (National Collegiate Athletic Association, 2019). However, to-date, few studies have examined women's collegiate volleyball season using accelerometer and related athlete monitoring strategies.

Broadly described, the field of athlete monitoring aims to quantify stresses that are incurred by athletes both inside and outside practice and competitions to enhance athletic performance, determine athlete readiness, and to mitigate injury risk (Coutts et al., 2018). The concept of athlete monitoring has always been a part of sport, however, the ways in which metrics are tracked has evolved over time. The athlete monitoring can be viewed in four domains: external workload, internal workload, perceptual wellbeing, and readiness (Gabbett et al., 2017). The external load refers to a player's locomotor movements (Halson, 2014; Gabbett, 2016). External load has historically been measured using metrics such as tonnage or duration, but more recently the use of GPS and accelerometers have become popular for tracking locomotion in practice and competition. Accelerometers provide more context to the movements undertaken by an athlete during sport when compared to measures such as duration. However, these accelerometers measure whole-body accelerations, therefore, direct measurement of specific body segment mechanical loads is limited (Ancillao et al., 2018).

Internal load refers to the physiological responses of players to external loads and can be determined using heart rate, biomarkers, ratings of perceived exertion (RPE), and session RPE (Foster et al., 2001; Halson, 2014; Bourdon et al., 2017; Gabbett et al., 2017). Perceptual wellness gives insight into how the athlete feels they are coping with training or life stresses and are typically collected using subjective wellness questionnaires (Saw et al., 2017). Lastly, athlete readiness examines whether an athlete is prepared to practice or compete and is determined through a combination of the previous factors (Gabbett et al., 2017).

Research in volleyball has focused on comparing objective external load measures such as the number of counter-movement jumps to subjective internal load measures like wellness scores or RPE during relatively short timeframes in professional men's leagues (Freitas et al., 2014; Debien et al., 2018; Duarte et al., 2019; Horta et al., 2019). A 2017 article by Vlantes and Readdy (2017) documented the loads of a single collegiate women's volleyball team during 15 games using accelerometer technology and RPE scores. This report provided a baseline of game workloads in collegiate volleyball, but did not include practices or athlete-perceived well-being. The NCAA's emphasis on student-athlete welfare and teams looking for a competitive advantage are reasons why many athlete monitoring measures are collected, however, from a research perspective, athlete monitoring is still in its infancy. As more sports and teams introduce accelerometers, the first step is to evaluate trends and add context of external and internal load measures in both practices and games throughout a competitive season.

Past and recent articles (Coutts, 2016; McLean et al., 2019; Impellizzeri et al., 2020) have highlighted the importance of methodical and thoughtful data collection and interpretation. Much of the current research in athlete monitoring has moved directly into studies which make broad inferences regarding injury, performance, and other athlete outcomes without first doing the work of understanding how different streams of data varying over an entire season as an entire team and by position group.

The aim of this study was two-fold. First, to contextualize accelerometer workloads, subjective wellness scores and RPE measures during games and practices over an entire collegiate women's volleyball season. The second aim was to explore differences between position groups in both sets of measures.

## Methods and Materials

### Participants

Data were collected from collegiate volleyball players (*n* = 11; 17 total athletes on the team) from a single National Collegiate Athletic Association (NCAA) Division I team. The team rostered 17 athletes, however, only 11 athletes were issued accelerometer devices. The 11 athletes were chosen based on who would be returning the following year. The participants mean age, height, body mass, and collegiate playing experience were 19.36 ± 1.27 years, 69.91 ± 3.88 cm, 64.04 ± 7.08 kg, and 1.91 ± 0.94 years, respectively. Within the squad, 4 players were outside or right side hitters (OH/RS), 3 players were libero/defensive specialists (L/DS), 2 were middle blockers (MB), and 2 were setters. The study protocol was approved by the university's institutional review board (IRB). Due to the practical setting in which measures were collected and the retrospective nature of data analysis, the University of Virginia IRB determined that participants were not required to provide informed consent (Protocol Number 2217).

### External Load

External workload was quantified through accelerometer units, with data collected from any session (training or game) in which a player participated. The units (Clearsky T6; Catapult Innovations, Melbourne, Australia), which incorporate a tri-axial accelerometer, gyroscope, and magnetometer, were placed on the back of players (between the scapulae) in a pocket sewn into a fitted Catapult Sports harness. The units were sampled at a rate of 100 Hz and athletes wore the same unit for the entire season. Devices from this company have shown “excellent” intradevice reliability with ICCs ranged from 0.77 (95% CI: 0.62–0.89) (very large) to 1.0 (95% CI: 0.99–1.0) (nearly perfect) (Nicolella et al., 2018).

**Figure 1:**
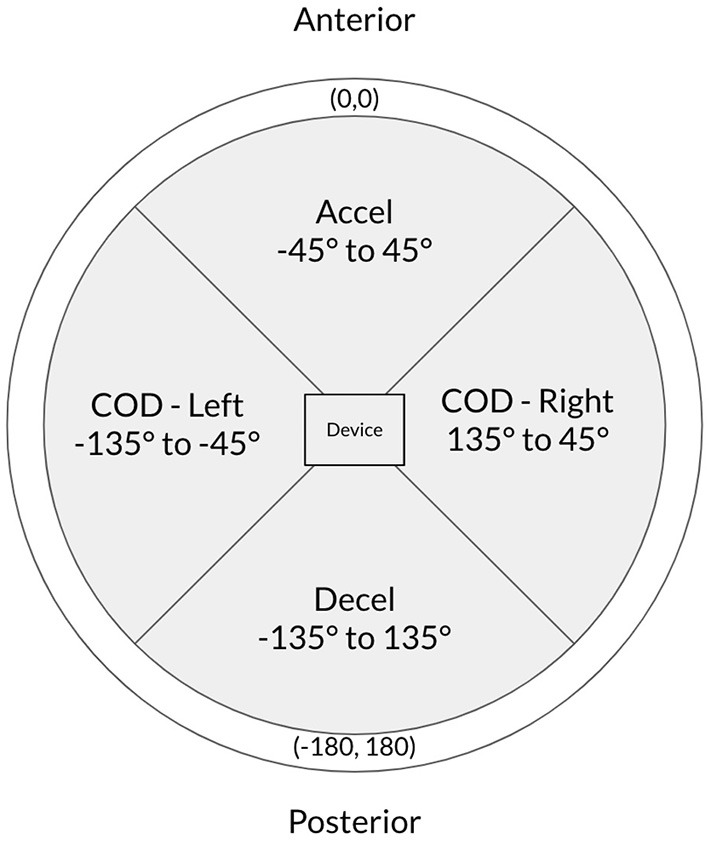
Directional graph of IMA measures.

After each session, the data were downloaded into a specialized analysis program (OpenField; Catapult Innovations, Melbourne, Australia—Version 1.22.2 Build #41409). Accelerometer variables and definitions are in Table 1 with a directional chart shown in Figure 1 (Spangler et al., 2018; Catapult Sports, n.d.).

**Table 1:** Accelerometer variable descriptions.

**Accelerometer variable**	**Description**
Playerload (PL)	The sum of the accelerations across all axes of the internal tri-axial accelerometer during movement. It takes into account the instantaneous rate of change of acceleration and divides it by a scaling factor (divided by 100). It can also be thought of as a cumulative acceleration load variable.
Inertial Movement Analysis (IMA)^∧^	A set of metrics that measures athlete micro-movements and direction regardless of athlete orientation using the tri-axial accelerometer and gyroscope. The measure graphically maps movements based on a clock model (Figure 1). The unit of measure is m/s.
Change of Direction (COD)	A count of the number of times the athlete changed direction as determined by the direction of the applied force and the orientation of the athlete.
Accelerations (accel)	A count of total IMA movements registered in an anterior vector (Figure 1).
Decelerations (decel)	A count of total IMA movements registered in a posterior vector (Figure 1).
Repeated High-Intensity Efforts (RHIE)	A count of the number of times the athlete completed 3 or more high-acceleration (≥2.79 m/s^−2^) movements with <21 sec of recovery between the efforts.
Jumps	Count of the number of times the athlete was in the air is > 320 ms; the average downward acceleration is at least ½ g, and the takeoff and landing peaks account for at least $\frac{1}{3}$ of the acceleration that such a jump would require
Low band	A jump registering 0 to 20 cm
Medium band	A jump registering >20 to 40 cm
High band	A jump registering >40 cm

∧ *IMA was not a specific variable used in the analysis, however, it is the basis other variables used and was important to add to this table*.

Data collection and management over the season were as follows:

- Practice sessions were full-team, coach-instructed sessions. Individual sessions, extra repetitions, pre-game walk-throughs, and strength and conditioning sessions were not included in the dataset.
- Players were “benched,” meaning their data was not collected during drill transitions, water breaks, or coaching instruction.
- Players not involved in a drill or rotated out during a large-sided drill were also “benched” in the collection system.
- Game data began with the start of the first set and ended with the conclusion of the final set. Players were “benched” when not on the court, during timeouts and challenge timeouts, and in between sets.

The season was divided into three time points: preseason (27 days), non-conference play (19 days), and conference play (64 days). Preseason covered the first 4 weeks of the season and included only practices. Non-conference play lasted 4 weeks and included weekend tournaments which were played on consecutive days with 1 day of the tournament consisting of two games. Lastly, conference play was the remaining 10 weeks of season and included, on average, four practices and two games per week. Games were typically played 1 day apart. Away competitions were played in the same weekend lending to travel weekends where the team would not travel home between away games. The collected timeframe was a total of 101 consecutive days in length. Within the timeframe, 55 practices and 30 games were captured. At the player-session level, this amounted to 770 total observations for analysis.

### Wellness Scores

Subjective measures of wellness were recorded through an app-based platform (AthleteReady, https://readyathleteapp.com) each morning before players arrived at the gym. Scores for fatigue, stress, muscle soreness, feeling healthy, and sleep quality were collected on a seven-point scale with higher scores being indicative of reduced wellness. Besides the numerical scale, athletes were shown emoji's that were descriptive of each wellness value. This wellness tracking platform was already being utilized by the team before the start of the study. To our knowledge, no other studies have utilized this specific subjective wellness platform, although, it is akin to other numerical scale-based wellness questionnaires (Saw et al., 2017). Within a sporting context and in a comparable age population, an ICC = 0.58 was calculated for subjective wellness questionnaires. According to the authors, this moderate reliability likely reflects variations in player responses from week to week, both in how they felt and normal variation in training loads (Gastin et al., 2013). Wellness scores were only included in the dataset on days in which there was also accelerometer data available (excluded data was <1% of total observations).

### RPE

RPE was used to capture subjective internal workload. Each player reported her RPE within 30 min of the end of the session using the Borg CR-10 scale (Borg, 1982; Foster et al., 1995; Uchida et al., 2014). Previous research indicates good test-retest reliability (ICC > 0.75) in RPE measures after exercises (Lamb et al., 1999). All players were educated on the use of the RPE scale at the beginning of the season and were instructed to rate their perceived effort for the whole session.

### Missing Data and Injury

Athletes sustaining season-ending injuries that precluded them from completing at least 50% of the season were excluded from analysis (*n* = 2, not included in the study participant count). This decision was made to remain in alignment with study aims, which is to contextualize an entire season of volleyball. Players who were modified for practice due to injury still had their data included, whereas, players who sustained a time-loss injury had their data removed during their convalescence period. If a player did not wear their accelerometer unit for reasons outside of injury, no data, including wellness scores, was captured for the session. Hardware issues, as detected by aberrant data, were also removed from the analysis. Additionally, there are two time periods of missing data due to logistical issues in data collection. The first time period was the 1st week [8 days, 11 practices (15%)] of preseason and the second was 6 days at the beginning of conference play [4 practices (6%) and 2 games (6%)]. Missing RPE data was imputed via a regression equation using accelerometer data based on previous research from our group (Kupperman and Hertel, 2020). In total, 10 days (~10% of data) was imputed in this manner.

### Statistical Analysis

Given the exploratory nature of this investigation to contextualize player workloads throughout a women's collegiate volleyball season, a pre-emptive decision was made to use primarily descriptive assessments. When objective comparison was needed, inferential statistics were utilized. An one-way analysis of variance (ANOVA) was used to determine whether there are any statistically significant differences between the means of the accelerometer, RPE, and wellness variables and different season time points. Where indicated, *t* tests were used to determine group difference at each time points (preseason, non-conference, conference) and also between session type (games vs. practices). Additionally, to examine the relationship between RPE and accelerometer variables, correlations were performed using a Pearson correlations coefficient (*r*). Correlations were evaluated using the following criteria: trivial: 0–0.10; small: 0.11–0.3; moderate: 0.31–0.50; large: 0.51–0.70; very large: 0.71–0.90; and almost perfect: 0.91–1.00. Analyses were completed in Python (version 3.8.0). Where applicable in figures, the mean and 95% confidence intervals (CI) are shown. The level of significance was set at *P* < 0.05.

## Results

Table 2 provides descriptive statistics of accelerometer and wellness measures for practices and games. For the accelerometer variables, the results show comparable means between practices and games; however, the standard deviations and ranges demonstrate notable variation in these metrics. Wellness measure and RPE averages are also similar across and the session types. From this season overview, it is clear that a further breakdown of the data is warranted from both a position and a seasonal trend standpoint.

**Table 2:** Mean and standard deviation of microsensor and wellness measures for all athletes per session type.

**Accelerometry measures**	**Practice (** ***n*** **=** **526)**		**Game (** ***n*** **=** **244)**	
	**Mean (SD)**	**Min/Max**	**Mean (SD)**	**Min/Max**
PL	388.3(128.5)	14.9/775.6	348.4(145.9)	14.9/1195.7
COD	247.5(121.7)	0.0/186.0	229.4(124.8)	0.0/858.0
Accels	93.6(46.9)	0.0/315.0	85.2(47.2)	0.0/334.0
Decels	94.8(52.9)	0.0/310.0	66.0(39.7)	0.0/343.0
Jumps	90.9(51.2)	0.0/258.0	81.1(49.8)	0.0/323.0
Low jumps	12.7(9.3)	0.0/54.0	11.9(9.5)	0.0/59.0
Medium jumps	46.5(50.0)	0.0/229.0	39.1(37.8)	0.0/229.0
High jumps	31.8(35.0)	0.0/146.0	30.0(33.6)	0.0/189.0
RHIE	4.2(4.6)	0.0/20.0	3.6(4.4)	0.0/20.0
**Wellness Measures**	**Practice (** ***n*** **=** **500)**		**Game (** ***n*** **=** **232)**	
	**Mean (SD)**	**Min/Max**	**Mean (SD)**	**Min/Max**
Fatigue	2.1(0.9)	1.0/6.0	2.0(0.9)	1.0/5.0
Muscle soreness	1.9(0.9)	1.0/5.0	2.0(0.9)	1.0/5.0
Feel Healthy	1.7(0.9)	1.0/5.0	1.8(0.9)	1.0/5.0
Stress	2.1(1.1)	1.0/6.0	2.1(1.0)	1.0/6.0

*The n equals the number of individual player observations in the dataset for each of the measurement categories*.

### Season Time Point Analysis

**Figure 2:**
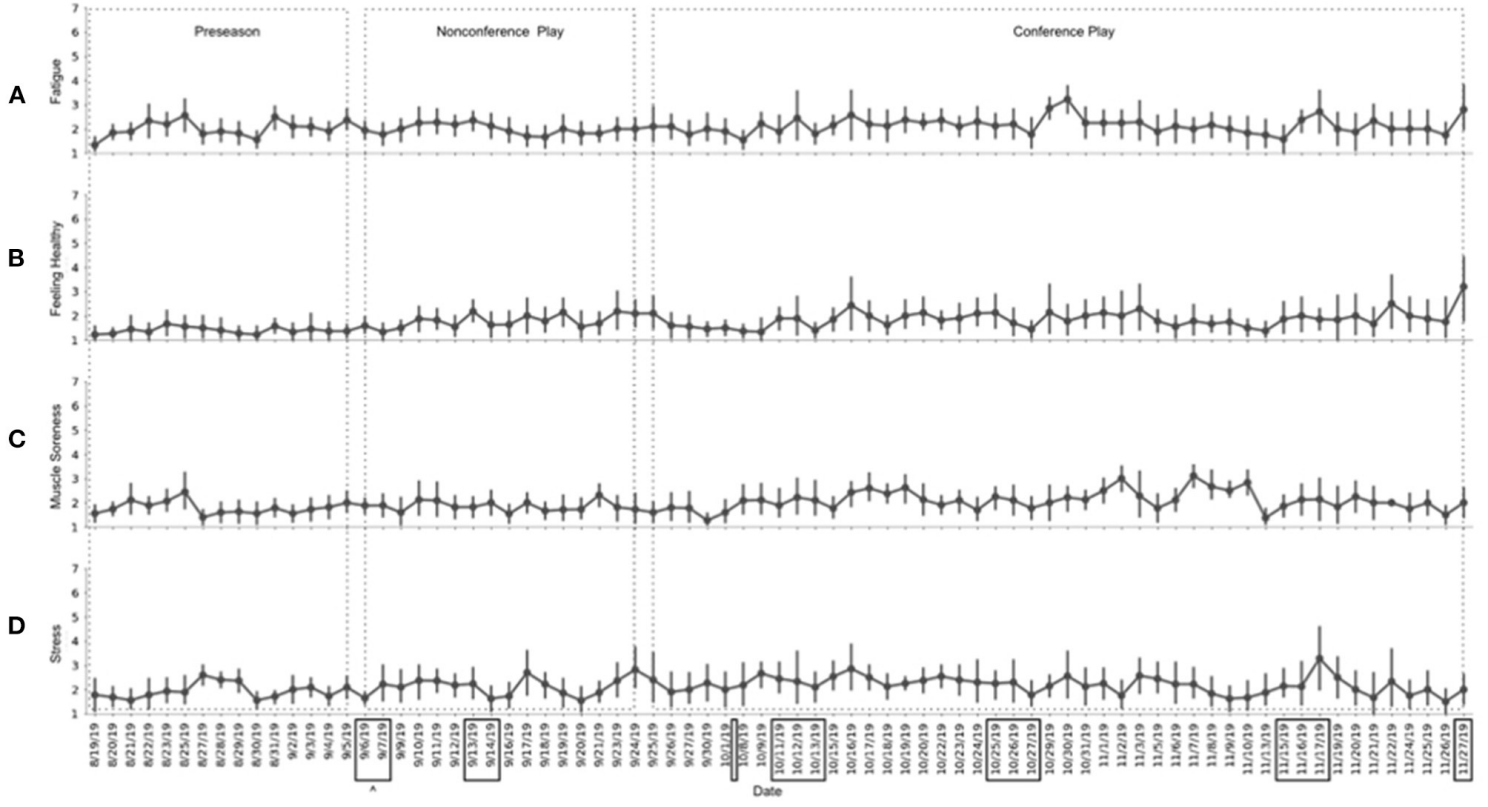
Average team wellness scores over the season. **(A)** Perceived fatigue values over the season; **(B)** Perceived feelings of being healthy values over the season; **(C)** Perceived muscle soreness values over the season; **(D)** Perceived stress values over the season. Point estimates represent the team mean for the day and the error bars represent 95% CI. ^∧^Boxes denote days of travel.

Wellness measures were analyzed by looking at team averages over the season. The results are shown in Figure 2. Across the entire season, statistically significant differences are noted between the season time points (stress: *F*_(2, 359)_ = 1.63, *p* = 0.002; muscle soreness: *F*_(2, 347)_, *p* = 0.002; feeling healthy: *F*_(2, 367)_, *p* = < 0.001). Stress, muscle soreness, and feeling healthy all had poorer ratings during conference play then compared to preseason and non-conference play (stress: *t*_(730)_ = −3.21, *p* = 0.001; muscle soreness: *t*_(730)_ = −3.36, *p* = 0.001; feeling healthy: *t*_(730)_ = −4.29, *p* < 0.0001). Upon inspection of Figure 2, it is clear there is an upward trend in fatigue during the month of October. A comparison of October to the rest of the season reveals a statistically significant difference in fatigue (*t*_(730)_ = −2.34, *p* = 0.02), stress (*t*_(730)_ = −3.15, *p* = 0.002) and muscle soreness (*t*_(730)_ = 0.021, *p* = 0.02) during this time.

**Figure 3:**
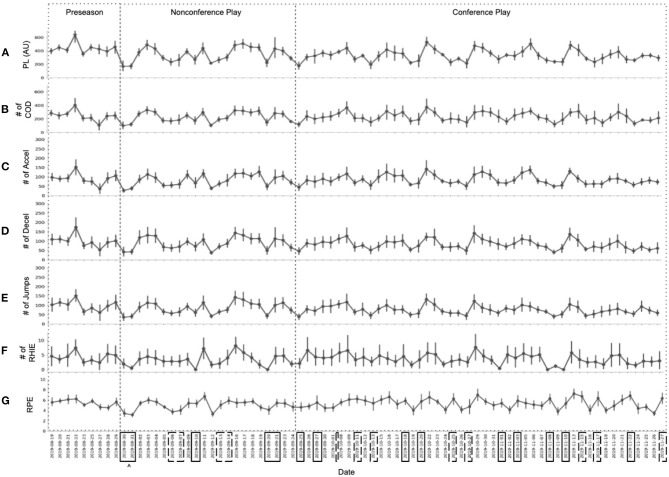
Average Team Accelerometer Totals Over the Season. **(A)** Average playerload (PL); **(B)** Average number of change of directions (COD); **(C)** Average number of accelerations (accel); **(D)** Average number of decelerations (decel); **(E)** Average number of jumps; **(F)** Average number of repeated high-intensity efforts (RHIE); **(G)** Average rating of perceived exertion (RPE). Point estimates represent the mean team value for the specific date with error bars showing the 95% CI. ^∧^Boxes around dates indicate values are from a game session with solid and dashed boxes denoting home and away games respectively.

Values for RPE over the season (Figure 3) do not appear to follow any seasonal trends like wellness measures demonstrate (*F*_(2, 166.9)_ = 0.30, *p* = 0.74). However, the highest team averages for RPE appear to coincide with peaks in accelerometer variable averages (Table 3). Figure 3 examines accelerometer variables over the season, it is apparent the variables follow similar trends to each other (distribution plots for accelerometer variables is available in Supplementary Figure 1). Most notably, average external load measures during games were less than during practices throughout the season (PL: *t*_(768)_ = −12.06, *p* < 0.0001; COD: *t*_(768)_ = −5.92, *p* < 0.0001; accel: *t*_(768)_ = −8.41, *p* < 0.0001; decel: *t*_(768)_ = −4.46, *p* < 0.0001; jumps: *t*_(768)_ = −8.40, *p* < 0.0001; RHIE: *t*_(768)_ = −5.24, *p* < 0.0001). For the most part, the 95% CIs overlap day-to-day indicating a sizable variation within the daily team averages.

**Table 3:** Correlation matrix between accelerometer variables and RPE.

		**PL**	**# of COD**	**# of Accel**	**# of Decel**	**# of Jumps**	**RPE**
PL	Pearson's r	–					
	*p*-value	–					
# of COD	Pearson's r	0.689^***^	–				
	*p*-value	<0.001	–				
# of Accel	Pearson's r	0.689^***^	0.792^***^	–			
	*p*-value	<0.001	<0.001	–			
# of Decel	Pearson's r	0.800	0.512	0.447^***^	–		
	*p*-value	<0.001	<0.001	<0.001	–		
# of Jumps	Pearson's r	0.736^***^	0.363^***^	0.479^***^	0.701^***^	–	
	*p*-value	<0.001	<0.001	<0.001	<0.001	–	
RPE	Pearson's r	0.486^***^	0.337^***^	0.311^***^	0.562^***^	0.446^***^	–
	*p*-value	<0.001	<0.001	<0.001	<0.001	<0.001	–

*** *p < 0.001*.

*PL, playerload; COD, change of direction; accel, accelerations; decel, decelerations; RPE, rating of perceived exertion*.

**Figure 4:**
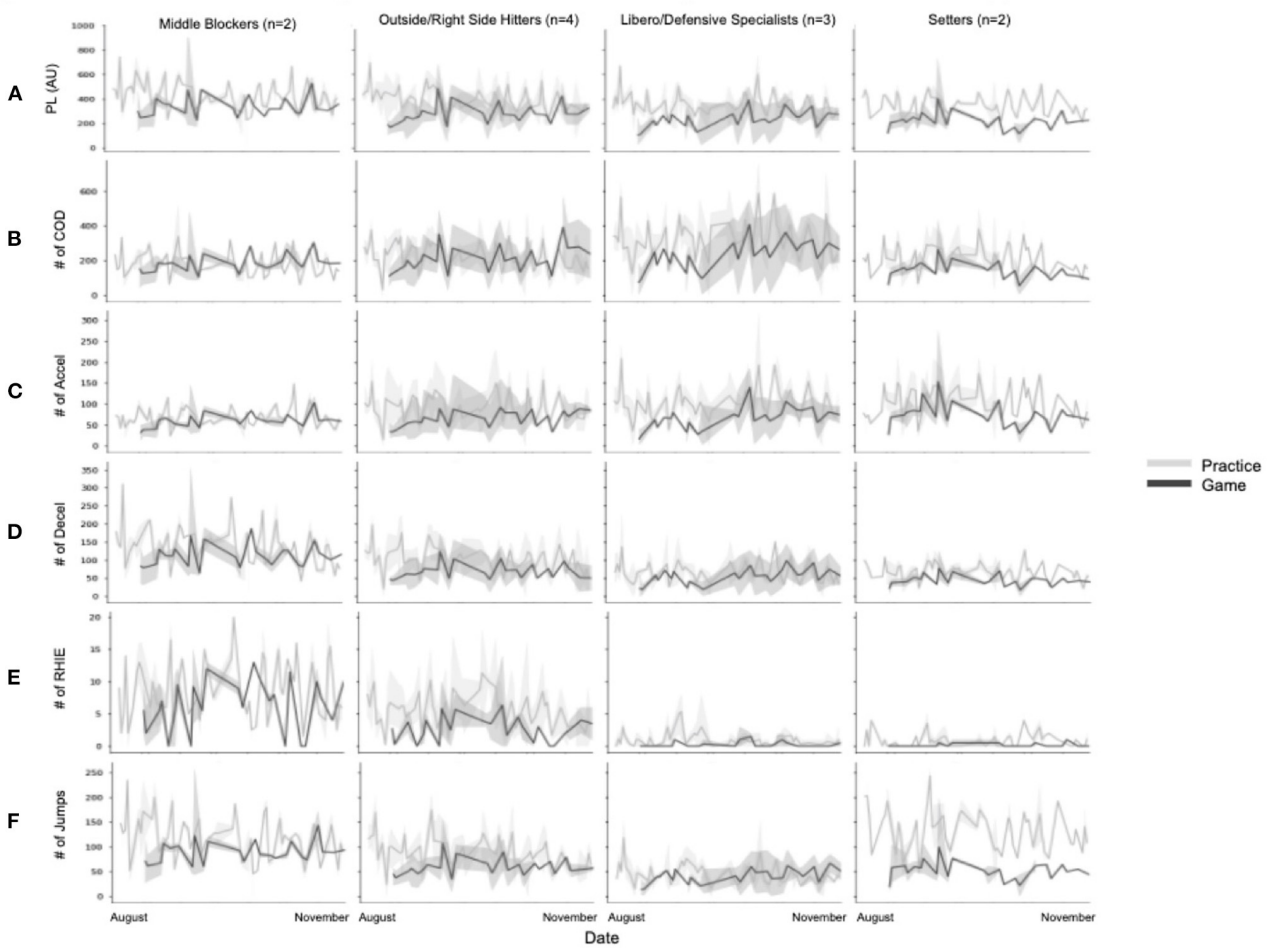
Mean accelerometry values over the season by position and session type. **(A)** Average playerload (PL); **(B)** Average number of change of directions (COD); **(C)** Average number of accelerations (accel); **(D)** Average number of decelerations (decel); **(E)** Average number of jumps; **(F)** Average number of repeated high-intensity efforts (RHIE). The shaded area represents 95% Cis.

To further understand this variation, the accelerometer measures tracked longitudinally across the season were divided into position-based graphs to explore differences between positions over a season (Figure 4). Broadly, this figure shows that each position group had unique trends for games and practices across all variables. It also highlights that specific variables may be more attuned to a specific group when attempting to understand the sport's workload demands. For example, on average, MBs had a greater number of RHIE for both games and practice compared to other positions (*t*_(768)_ = −14.22. *p* < 0.0001). Furthermore, for practices, the setter and MB groups had notably higher average total jumps than the L/DS and OH/RS groups (*t*_(768)_ = −11.87, *p* < 0.001). Also, of note, there was remarkable variation in both the L/DS and OH/RS groups for the variables of COD and accel, specifically during game sessions.

### Positional Differences

**Figure 5:**
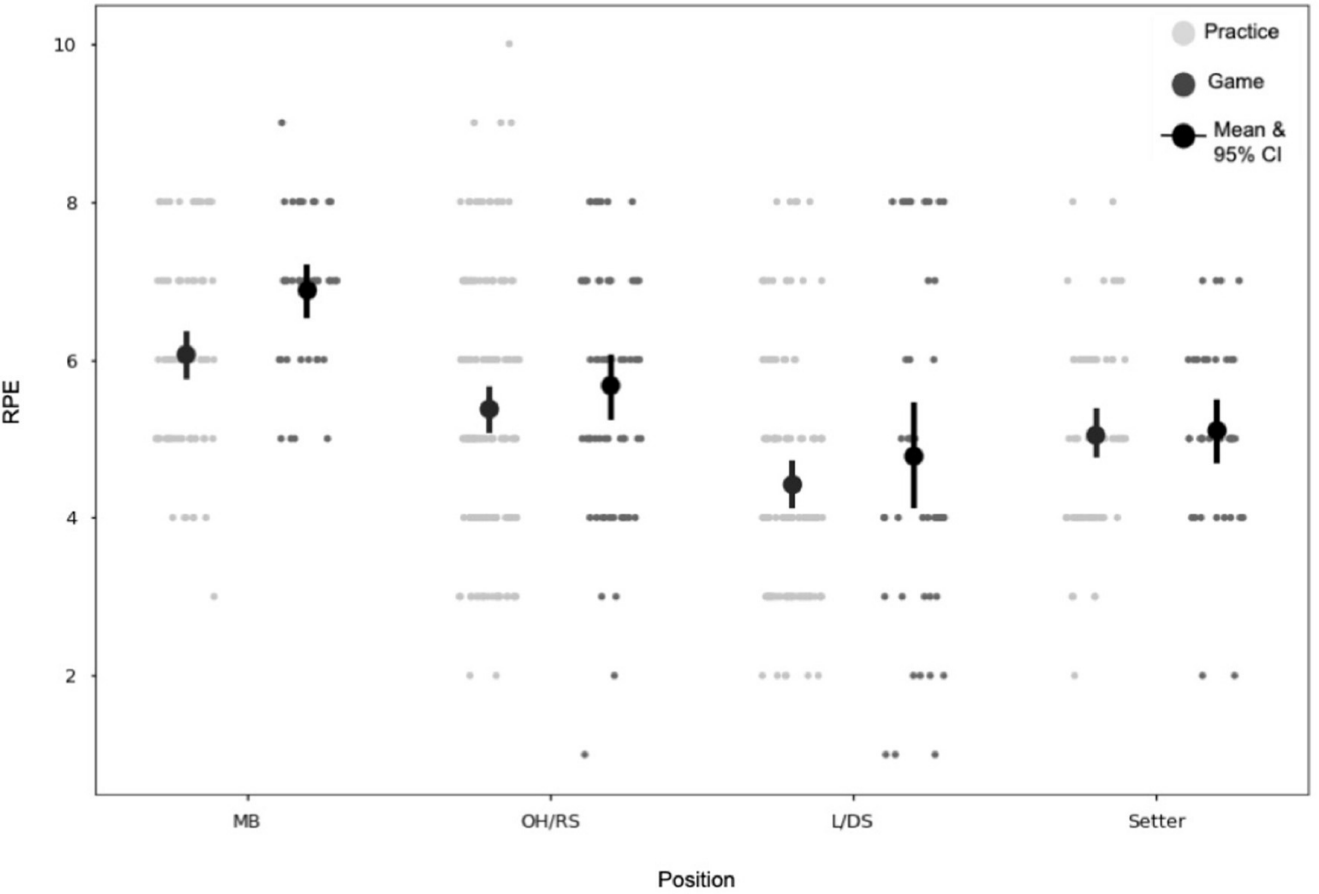
RPE by position and session type. Each point on the graph represents an individual player-session. MB, middle blocker; OH/RS, outside hitter/right side hitter; L/DS, libero/defensive specialist; RPE, rating of perceived exertion.

**Figure 6:**
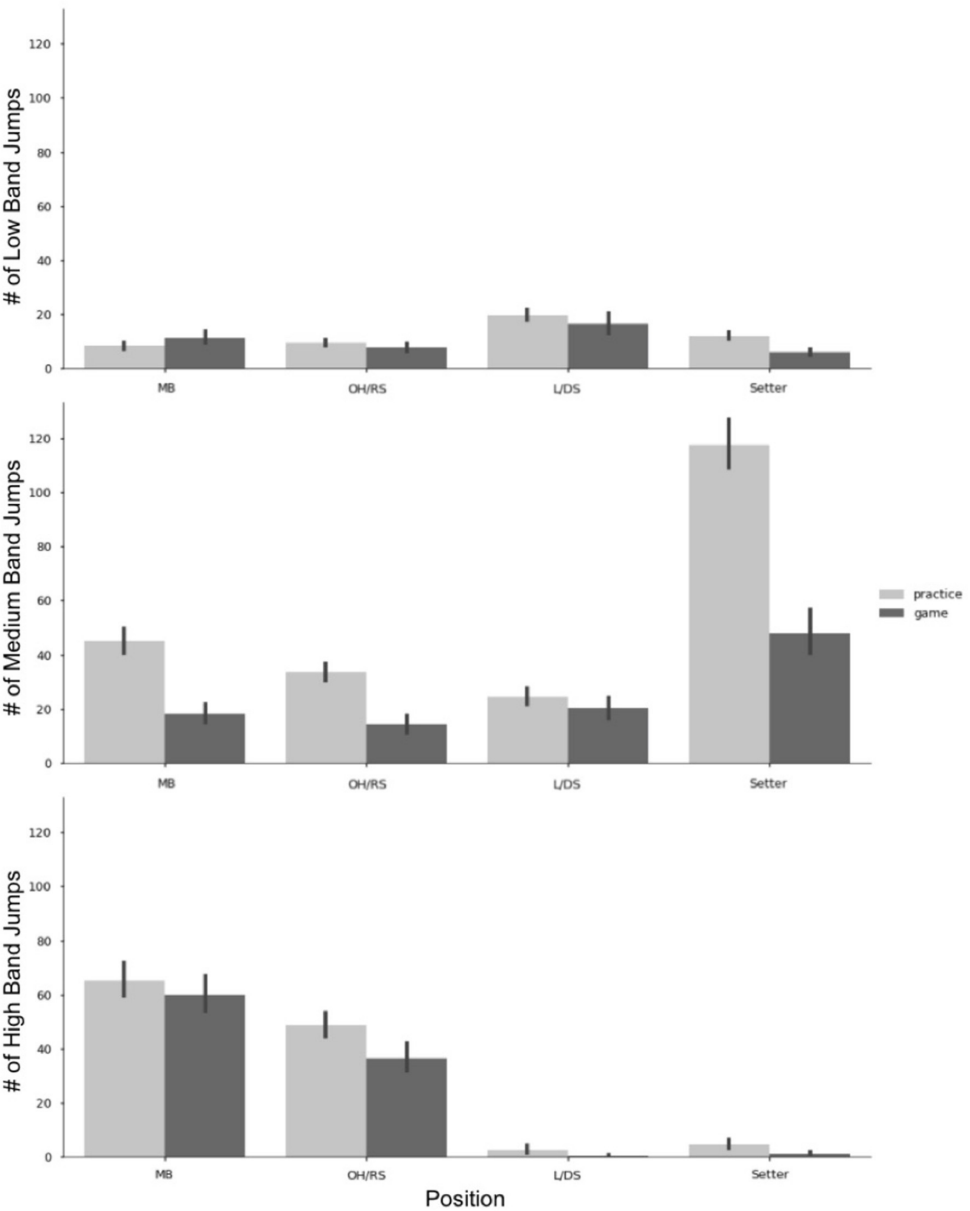
Jump counts by band, position, and game or practice. Error Bars represent 95% CI. MB, middle blocker; OH/RS, outside hitter/right side hitter; L/DS, libero/defensive specialist.

The most noteworthy findings of this analysis are in further understanding the differences between the position groups' external workload data. Figures 5, 6 show the average and 95% CI for accelerometer and RPE variables between both positions and practices and games across the four different position groups. For all positions, practice averages for all measures were greater for practices than for games. For MB, RHIE, decels had overlapping 95% CI between practices and games, whereas COD and jump counts confidence intervals overlapped for the OH/RS and L/DS position groups, respectively. Average RPE values across games and practices were similar for OH/RS, L/DS, and setter groups. The MB position group had higher RPE averages than the other groups for both games and practices (*t*_(608)_ = −8.16, *p* < 0.0001) and within the group game averages were notably greater than practice averages (*t*_(117)_ = 3.67, *p* = 0.0004) (Figure 5).

As described in Table 1, jump counts can be further broken down into low, medium, and high bands based on jump height. Figure 6 illustrates the differences between these jump bands in each position for both practice and games. Low band jumps had the least number of jumps for the team overall but were the band in which the L/DS group accumulated the highest number of jumps. For setters, most of their jumps were accumulated within the medium band, with over double the amount of medium band jumps in practices vs. games. Similarly, MB and OH/RS had approximately twice as many medium band jumps in a practice than they did in games. In the MB and OH/RS position groups, jumps were concentrated in the high band, with similar averages among practices and games.

**Figure 7:**
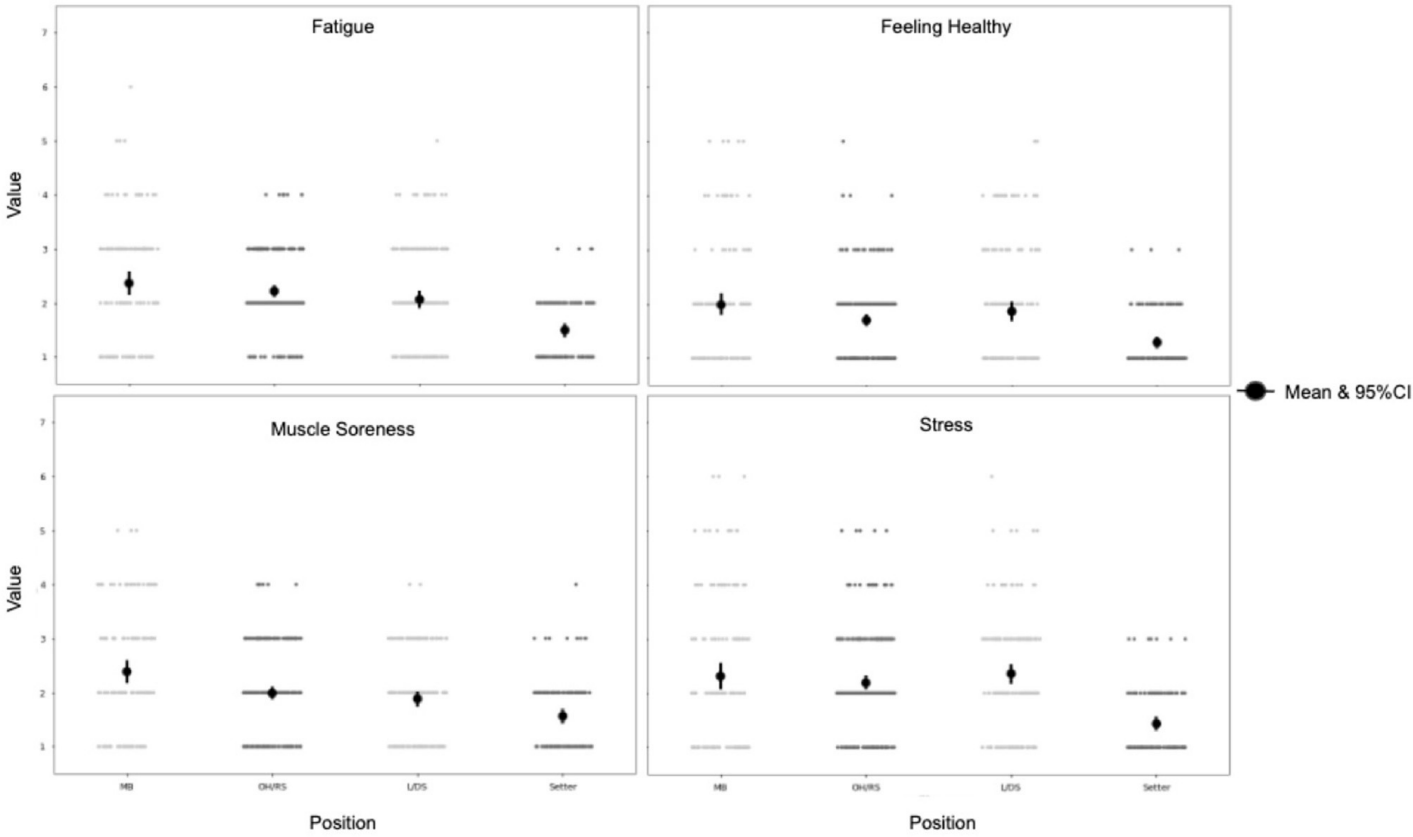
Wellness measures by position. Each point on the graph represents an individual player-session. A value of 1 indicates increased wellness, whereas a value of 7 indicates decreased wellness. MB, middle blocker; OH/RS, outside hitter/right side hitter; L/DS, libero/defensive specialist.

For wellness measures, across all positions, the season averages were between 2 and 3 out of 7 possible points, with the higher number being a worse perception of wellness (Figure 7). Setters had, on average, the lowest wellness values for fatigue, stress, muscle soreness, sleep quality, and feeling healthy. Whereas, the MB group had the highest point estimate for all wellness measures, however, the difference between these two groups is approximately 1-point (on the 7 point scale) for any given value. The OH/RS and L/DS groups had the most similar point estimates for all five wellness values.

## Discussion

This study provides an initial understanding of athlete monitoring variables in women's collegiate volleyball over a season. Overall, we found that while there were variations throughout the season in both wellness and accelerometer measures, as a whole, the team averages remained consistent. With the additional context of travel schedule, volleyball game play, and position groups, more striking patterns within the data emerged.

### Season Time Points

Other studies have documented that travel, specifically congested travel schedules, can impact wellness values (Conte et al., 2018; Rabbani et al., 2018). The month of October contained the most travel, and during this month, we saw an increase and subsequent plateau in all wellness values (poorer outcomes). Team travel during conference play usually included three nights away and traveling to two different opponents. Travel could include flight and bus rides up to 8 h in length. During November, fatigue remains elevated, most likely due to a combination of nearing the end of the season and an increase in academic work before final exams. The poorer outcomes for stress, muscle soreness, and feeling healthy during conference play vs. preseason and non-conference play could be attributed to the intensity of the game schedule and also the cumulative effects of a season.

Tracking wellness values can be useful to monitor internal loads from demands that incur from both in and out of sport activities. While the primary purpose of this study was to examine trends over a season, modifying training demands based on wellness may lend itself to more optimal recovery, especially during times of congested travel or known periods of stress, such as academic exams.

Accelerometer variables demonstrated notable trends over the volleyball season. The most notable, a possibly counterintuitive trend, is lower team averages for these variables during games compared to practices. Based on previous studies, we typically assume that, on average, game days require a greater physical demand than practice on athletes (Henderson et al., 2015; Gentles et al., 2018). However, to date, much of the longitudinal athlete monitoring research has been conducted in sports such as soccer, where the game is divided into defined time periods (halves, quarters,…), and player substitutions are relatively infrequent. On the other hand, volleyball matches are constructed into smaller sets based on points with player substitutions being cyclic and occurring with the majority of players. For example, on most collegiate teams, the MB plays three rotations in the front row and then is substituted out for an L/DS in the three back row rotations. The team's libero is a specialized position with different substitution rules and plays nearly all six rotations for an entire set. Additionally, a setter could play three or six rotations depending on the offensive strategy being deployed. Ultimately, there can be a patterned difference between athletes playing 50 or 100% of a set, which can have significant implications for the accelerometer measures. Also, since matches at the NCAA level are played best out of five sets, the total duration of the game can vary up to 30+ min. Understanding the rotational nature of volleyball, varied time domains, and that there are usually more athletes at practice than those who play in the games explains why game session external load measures were less than during practices.

Athlete monitoring is often used by teams to enhance athletic performance. Discovering that game external demands were less than what is being accumulated in practice, the question becomes: Should practices be altered to more closely mimic game demands? Theoretically, due to injury, substitution restrictions, or opponent, a player could be required to play all six rotations at any time. If only conditioned to play their usual 50% of the game, going to 100% could prove challenging. Answering these questions is not the aim of this paper, but in exploring season-long trends, these are the practical athlete monitoring questions that arose.

### Positional

**Figure 8:**
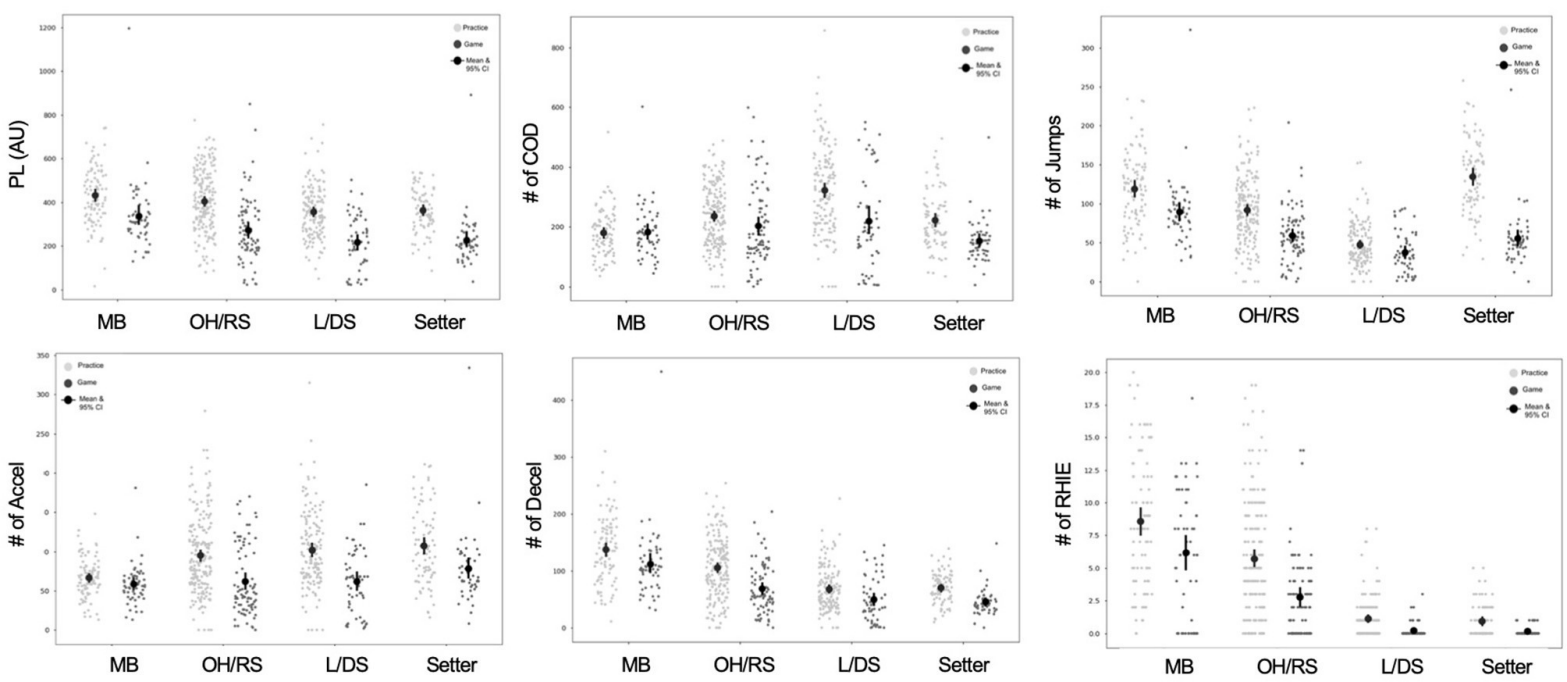
Accelerometry variables by position and session type. Each point on the graph represents an individual player-session. MB, middle blocker; OH/RS, outside hitter/right side hitter; L/DS, libero/defensive specialist; PL, playerload; COD, change of direction; accel, accelerations; decel, decelerations; RHIE, repeated high intensity efforts.

The variation within the daily averages of the accelerometer measures points to differences on a more individual player level. Examining measures by positional groups revealed more insights into the external demands of volleyball. Historically in volleyball jump counts have been the primary way to assess physical demands (Charlton et al., 2017; Lima et al., 2019); however, as Figure 6 shows, jumps are only pertinent to front row players and setters. Variables, such as PL and COD, provide a more accurate representation of the physical demands of back row players. Additionally, understanding of the front row positional demands can be enhanced with the addition of the number of decels and RHIE completed in a session. While Figure 8 shows variation within these measures, further work to understand where these loads are accumulated may be useful in planning and modifying training programs and also with return-to-sport protocols after injury (Gabbett, 2019; Taberner et al., 2019).

While the device used to collect accelerometer data did not have the ability to calculate exact jump height, it did classify jumps into low, medium, and high bands. In the low band, the majority of jumps occur for L/DS while these positions are not typically jumping positions, jumps in the low band could be accrued through serving, exaggerated shuffling or diving motions, and team celebration after a point scored. The setter group had the most surprising findings around jump counts. The majority of setter jumps occur in the medium band and can mostly be attributed to the motion of jump setting. Similar to the findings of Vlantes and Readdy (2017), the setter accumulated the most jumps of any position in a game. On a team-level, there is a setter present on the court the vast majority of the game, so the offensive system (e.g., 5–1 or 6–2) yields similar results. For player-level analysis, the system should be taken into account as in a 5–1 a single setter is accruing the jumps, whereas in a 6–2, 2 setters are splitting the total jumps.

Another surprising feature of Figure 6 revealed that on average, setters could accrue three times as many medium band jumps in a practice than in a game. We speculate that this occurs for two reasons, first refers back to the previous discussion of the rotational nature of volleyball. In a game, a setter may only be in play 50% of the time compared to practice, where they may constantly be setting for drills. Secondly, there are fewer setters on a team than hitters. Therefore, setters would need to set two to three times as many balls for the hitters to complete their number of desired repetitions. More data is needed to understand the physiological and biomechanical impacts of medium band jumps on musculoskeletal tissue, so we cannot speculate if this imbalance in jump counts between practice and games is detrimental to setters. Unsurprisingly, MB, and OH/RS accumulate the majority of their jumps in the high band, which can be attributed to attacking and blocking, the primary objectives of these positions.

The wellness measures broken down into position groups do not show any major differences in overall averages, but Figure 7 does highlight the difference in distribution between groups. These differences may be due to unequal numbers in each group, causing unintentional skewness. A larger sample size is needed to understand if there indeed are differences in wellness values between position groups, and a comparison to external load data is needed to understand if there is an impact on object physical demand and subject physical well-being.

### RPE

Other studies have documented that RPE and session RPE (RPE multiplied by the session duration in minutes) are correlated with objective workload measures (Casamichana et al., 2013). The current study found a large strength of association between RPE and the measured accelerometer variables (Table 3). Practically, this demonstrates RPE may be a useful tool in monitoring workloads in the absence of accelerometer technology.

### Limitations

As with any retrospective, in-the-field, data collection and analysis there are limitations to the results. First and foremost, this data was captured on a single team and specific trends may be unique to this team and not extend perfectly to other collegiate volleyball teams. Data collection occurred during the team's first season of using wearable sensors. Practically a methodical approach was taken to first collect and understand the data before making gross changes to training regiments. Secondly, the dataset does not include workload accumulated in individual session or weight room sessions. The ability to track athletes in all areas of training is something future research should strive to accomplish. Thirdly, there was occasional missing data for individual athletes and time periods of missing data for the entire team due to logistical issues. Even with these missing data, there was still an abundance of player-session observations to evaluate trends. Lastly, given wellness scores were collected for practical team monitoring and designed before this study was conducted, comparing the results to other studies is challenging.

### Considerations for Future Research

Collecting and contextualizing an entire sporting season in regards to workload and wellness outcomes allows for more introspection when selecting appropriate analyses for longitudinal data. Mean comparison tests often fail to demonstrate the variance in the data and neglect to capture within person processes. By first visualizing and describing the data, the strategies for testing longitudinal hypotheses become more apparent (Bolger and Laurenceau, 2013). While the team averages did not vary greatly over the season, the 95% CIs reveal between athlete and between position group variation, which suggests further analysis should take clustering factors and within person analysis into account when conducting outcomes-based research (Clarke, 2008; Hopkins et al., 2009; Curran and Bauer, 2011). Additionally, the trends noted around different time points in the season and during congested travel, highlight the importance of maintaining a longitudinal framework when applying inferential and model-based statistics (Bittencourt et al., 2016).

## Practical Applications

We observed notable trends in both wellness and accelerometer data throughout the season, showing that these measures have obvious practical implications for practice design and future investigation into their impact on athlete outcomes. This study also shows that accelerometer variables beyond that of jump count are useful in a volleyball setting. Variables such as PL and COD can be more broadly reflect activity in all positions in volleyball leading to a better understanding of the physical demands of the team as whole. Finally, we highlighted the importance of sport-specific context (i.e., the rotational nature of volleyball games) in the analysis of external load data.

## Conclusion

In summary, we quantified, for the first time, the season-long practice and game demands during a competitive season in women's collegiate volleyball. For wellness measures, we saw trends in team values based on travel and time in season, whereas for accelerometer variables, longitudinal trends noted differences between practices and games. Importantly, although we report team averages for workload did not vary greatly, there was notable variation within each time point. This led to evaluation of positional differences, which revealed trends, specifically in the number jumps accumulated in each band. Cumulatively, this data suggests athlete monitoring can be used to understand the demands of volleyball and used in the future to enhance practice and recovery day design to optimize athlete well-being.

## Data Availability Statement

The datasets presented in this article are not readily available because human subjects data is protected beyond research team. Requests to access the datasets should be directed to nak5dy@virginia.edu.

## Ethics Statement

The studies involving human participants were reviewed and approved by University of Virginia Social and Behavioral Science Institutional Review Board. Written informed consent for participation was not required for this study in accordance with the national legislation and the institutional requirements.

## Author Contributions

NK, MC, and JH contributed to study design and implementation. NK carried out all data collection and analysis. All authors contributed to the interpretation and discussion of results. Additionally, all authors contributed to the editing of the article and approved the submitted version.

## Conflict of Interest

The authors declare that the research was conducted in the absence of any commercial or financial relationships that could be construed as a potential conflict of interest.

## Publisher's Note

All claims expressed in this article are solely those of the authors and do not necessarily represent those of their affiliated organizations, or those of the publisher, the editors and the reviewers. Any product that may be evaluated in this article, or claim that may be made by its manufacturer, is not guaranteed or endorsed by the publisher.
