# Supplementary figures and images for: Quantification of Workload and Wellness Measures in a Women's Collegiate Volleyball Season
Source: Front Sports Act Living. 2021 Aug 6;3:702419. doi: 10.3389/fspor.2021.702419 (PMC8377283; doi:10.3389/fspor.2021.702419)

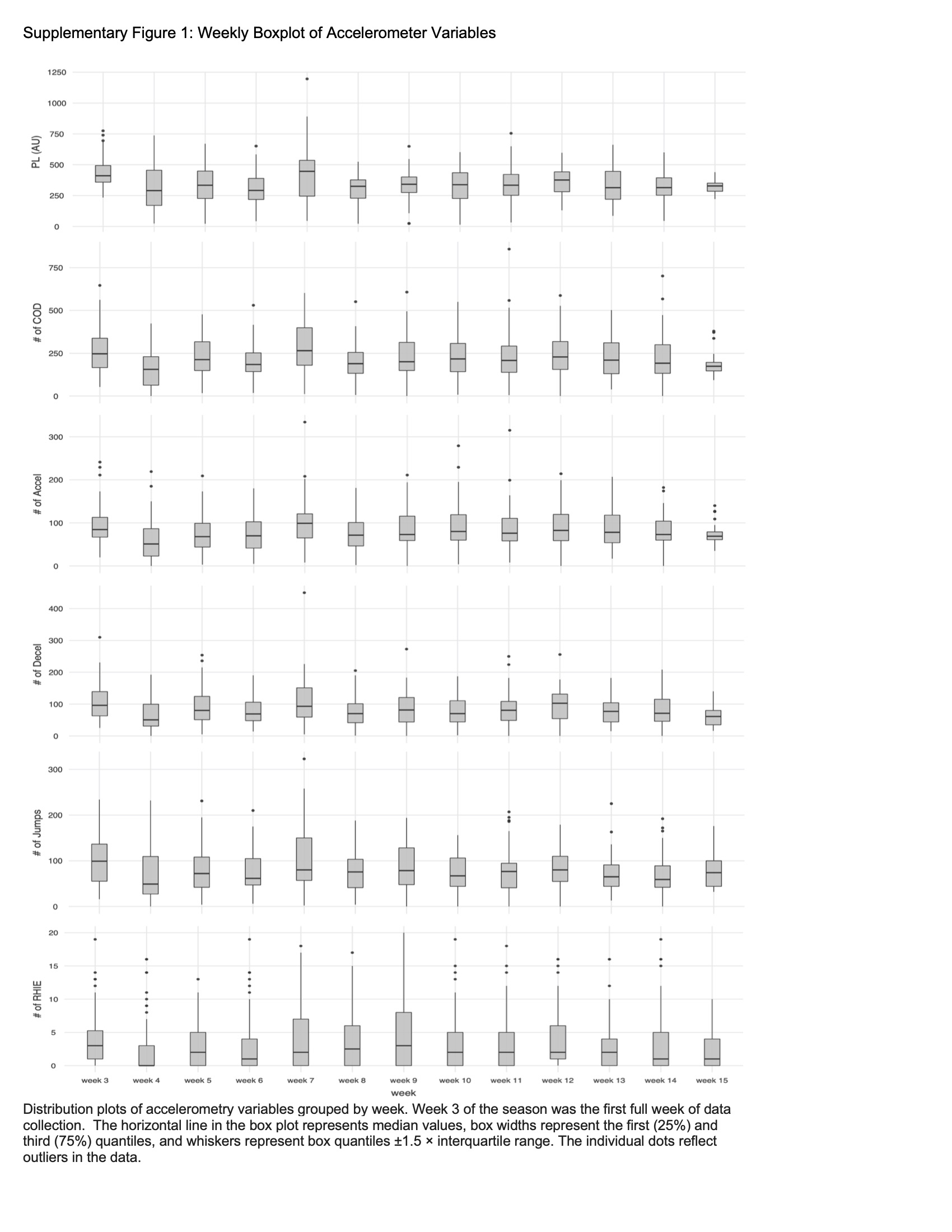

Supplement: Supplementary file 1 [file Image_1.JPEG]
